# Effects of Metformin, Pioglitazone, and Silymarin Treatment on Non-Alcoholic Fatty Liver Disease: A Randomized Controlled Pilot Study

**DOI:** 10.5812/hepatmon.6099

**Published:** 2012-08-03

**Authors:** Ali Akbar Hajiaghamohammadi, Amir Ziaee, Sonia Oveisi, Homa Masroor

**Affiliations:** 1 Metabolic Disease Research Center, Qazvin University of Medical Science, Qazvin, IR Iran

**Keywords:** Metformin, Pioglitazone, Silymarin, Cardiovascular System, Non-Alcoholic Fatty Liver Disease

## Abstract

**Background:**

Nonalcoholic fatty liver disease (NAFLD) is one of the most common reasons of enzyme increase in liver. In About 10 percent of patients with NAFLD, the disease progresses toward Non Alcoholic Steatohepatitis (NASH) and about one third of them may progress toward cirrhosis, liver dysfunction, and even hepatocellular carcinoma.

**Objectives:**

According to high prevalence of NAFLD and the fact that there is no consensus on treatment of this disease, the aim of this study was to assess the effects of metformin, pioglitazone, and silymarin on treatment of NAFLD.

**Patients and Methods:**

Sixty six patients with NAFLD who were presented in the Endocrinology and Metabolism clinic of Boo’ali Hospital, Qazvin, Iran, were assigned randomly into three groups (n = 22). First group was treated by pioglitazone 15 mg/d, second group by metformin 500 mg/d, and third group by silymarin 140 mg/d. All patients underwent clinical and biochemical evaluations including weight, fasting blood sugar (FBS), lipid profiles, body mass index (BMI), aspartate aminotransferase (AST ), alanine aminotransferase (ALT), and serum insulin levels in pre- and post-intervention after eight-week follow up.

**Results:**

Before the treatment there was no significant difference between three groups with respect to average age, BMI and gender, FBS, lipid profile, AST, ALT, serum insulin level, and Homeostasis Model Assessment (HOMA) index for insulin resistance. After the intervention, a significant reduction was observed in average amount of FBS, lipid profile, ALT, AST, serum insulin level and HOMA index in three groups (P < 0.01). The most reduction in average FBS, TG, serum insulin level, and HOMA index was observed in pioglitazone group, the most reduction in average amount of cholesterol was seen in metformin group, and the most decrease in average amount of AST and ALT occurred in silymarin group.

**Conclusions:**

These results suggest that all drugs are beneficial in improving biochemical indices in patients with NAFLD. Changes in AST and ALT in silymarin group were demonstrated more than that in other groups and the average difference between changes was significant between silymarin and metformin groups.

## 1. Background

Nonalcoholic fatty liver Disease (NAFLD) occurs in non-alcoholic persons or in whom with little consumption. It is estimated that 20-40 percent of population of west countries and 5 to 30 percent of population in Asia and Oceania are afflicted with this disease (1, 2). The histological characteristic of NAFLD is accumulation of macro vesicular lipid similar to liver disease due to chronic consumption of alcohol. Fatty liver and steatohepatitis are two histological conditions for this disease. Liver histology in this disease is indistinguishable from alcoholic hepatitis and includes balloon degeneration, hepatocytes necrosis, and fibrosis. Currently there are no comprehensive and acceptable staging and grading system for this disease. Pathogenesis of NASH is not well-understood but often the presence of two damages from which the first damage leads to accumulation of lipids in liver and steatosis and the second damage to inflammation and fibrosis is an accepted mechanism. Resistance to insulin is likely the reason for the first damage in most patients, while oxidative stress and lipid peroxidation or damage by inflammatory cytokines are considered as responsible for the second damage (3). In regard to the treatments of NAFLD, currently there is no consensus, similar to other aspects of this disease. One of the treatments proposed is active essence of silybun marianum plant known as silymarin. Four different mechanisms of action in silymarin and silibinin are recognized: (1) as antioxidants, scavengers, and regulators of intracellular content of glutathione; (2) as cell membrane stabilizers and permeability regulators; (3) as promoters of ribosomal RNA synthesis, stimulating liver regeneration; and (4) as inhibitors of stellate hepatocyte transformation into myofibroblasts. Metformin is a biguanide, approved as a hypoglycemic therapy in patients with type 2 diabetes mellitus. This agent improves sensitivity to insulin by reducing hepatic glucose production, reducing lipolysis in adipose tissue, increasing peripheral glucose uptake by liver, skeletal muscle and adipose tissue, and inhibiting intestinal glucose absorption (4, 5, 6, 7, 8). Pioglitazone is an orally administered insulin-sensitizing thiazolidinedione agent developed for the treatment of type two diabetes mellitus. Pioglitazone activates nuclear PPAR-γ, which leads to increased transcription of genes encoding various proteins regulating glucose and lipid metabolisms (8, 9, 10).

## 2. Objectives

The aim of this study was to compare effectiveness of silymarin, pioglitazone, and metformin in improving insulin sensitivity and some biochemical markers in NAFLD.

## 3. Patients and Methods 

### 3.1. Participants

**Figure 1:**
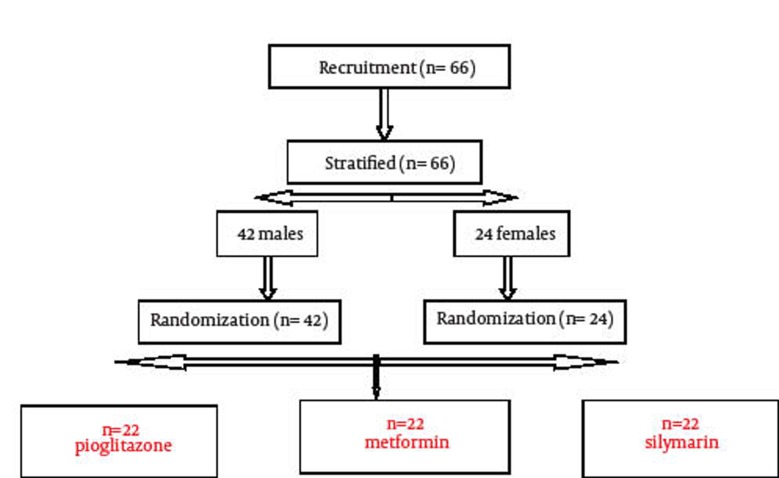
The Trial Profile of Patients Who Completed Follow Up

This study was conducted in Qazvin, Iran. Sixty six patients (males = 42 and females = 24) who were presented in Endocrinology and Metabolism clinic of Boo’ali Hospital, Qazvin University of Medical Sciences from 2010 to 2011 were studied. Patients suffered from NAFLD (Mean age = 32.62 years, SD = 6.4 years) and their trial profileis shown in Figure 1. Some characteristics and parameters of participants are shown in Table 1. All participants provided written informed consent before enrolment and all responses were kept confidential. The ethics review board of Qazvin University of Medical Science approved the study. Inclusion criteria were infliction with NAFLD Confirmed by performing liver sonography and increased levels of liver enzymes AST and ALT. All tests and sonographies were conducted in one center. In addition to obtaining complete history on taking alcohol and drugs, the tests related to autoimmune hepatitis and virus markers were also applied for all patients. Patients having history of alcohol consumption, diabetes, chronic liver disease, use of drugs such as statin, fibrate, NSAID, and those with positive results for tests of autoimmune hepatitis and virus markers (HBS antigen. HCV antibody) were excluded from the study.

**Table 1:** Characteristics and Some Parameters of 66 Participants at Base Line

	**Mean ± SD**	**Minimum**	**Maximum**
Age, y	32.62 ± 6.40	22	44
Weight, kg	76.95 ± 9.25	62	96
FBS a, mmol/L	95.50 ± 6.58	85	109
TG a, mg/dL	251.57 ± 53.25	180	386
CHOL a, mg/dL	193.45 ± 34.12	137	275
AST a, IU/L	55.31 ± 10.49	39	79
ALT a, IU/L	78.18 ± 19.09	51	120
Insulin level, mmol/L	14.18 ± 3.80	9.3	24.4
HOMA-IR a	2.88 ± 92	1	2
BMI a, kg/m ^2^	27.44 ± 1.69	24.61	32.91

^a^Abbreviations: ALT, alanine aminotransferase; AST, aspartate aminotransferase; BMI, body mass index; CHOL, cholesterol; FBS, fasting blood sugar; HOMA-IR, homeostasis model assessment index for insulin resistance; TG, triglyceride

### 3.2. Procedure

First we categorized patients in two groups (24 females and 42 males) and randomly allocated patients into three intervention groups using 22 blocks A, B, C (ABC, ACB, etc.) of Balanced Blocked Randomization method. Group one was treated by pioglitazone 15 mg/day, group two by metformin 500 mg/d, and group three by silymarin 140 mg/d. All drugs were administered in the form of pills. Patients in each group took one pill daily for two months. Serum insulin assessment was performed by MONOBAND kits manufactured in USA. Other biochemical elements were assayed PARS-AZMOON manufactured DIASYS Germany Company. In order to compare effects of drugs, the weight, FBS, serum Insulin level, serum cholesterol and triglyceride levels, AST, ALT, Body Mass Index (BMI), and Homeostasis Model Assessment index for Insulin Resistance (HOMA-IR) were measured in three groups before and after drug administration.

### 3.3. Statistical Analysis

After data collection, findings were formulated as statistical tables and numerical indices. Analyses involving these measures consisted of repeated measures MANOVA using pre- and post-intervention FBS, lipid profiles, Insulin, AST, ALT, HOMA index, and BMI in three groups. Probability value less than 0.05 was considered as statistically significant.

## 4. Results

Sixty six subjects completed the study (22 in each study group). We first assessed distribution of variables before using parametric analyses and results showed that all of them were normal. None of patients withdraw from study during research. 14 males (63.4%) and eight females (36.6%) were studied in each of three groups of pioglitazone, metformin, and silymarin. Average ages for pioglitazone, metformin, and silymarin groups were 33.4 ±6.6, 32.5± 6.5 and 33.5± 6.3 years, respectively. Comparison of characteristics and some parameters of participants before and after treatment with pioglitazone, metformin, and silymarin are shown in Table 2. We were also interested in clinical significance of treatment. We used cut off 100 for FBS, 150 for TG, 40 for ALT and AST, 2.5 for HOMA-Index, and 200 for cholesterol (Table 3). Strength of treatment effects in three groups of intervention has been shown is Table 4.

**Table 2:** Repeated Measures MANOVA in Three Intervention Group (Metformin, Pioglitazone, Silymarin) After two Months Follows Up

	**Pioglitazone, Mean ± SD**		**Metformin, Mean ± SD**		**Silymarin, Mean ± SD**		**F (Time Treatment)**	**P value**
	**Before**	**After**	**Before**	**After**	**Before**	**After**		
Weight, kg	76.95 ± 9.17	77.82 ± 9.32	76.9 ± 9.52	75.46 ± 9.9	77.00 ± 9.50	77.09 ± 9.52	46.127	0.001
BMI a, kg/m ^2^	27.36 ± 1.65	27.67 ± 1.77	27.53 ± 1.85	27.00 ± 1.98	27.44 ± 1.65	27.48 ± 1.70	43.93	0.001
FBS a, mmol/L	95.45 ± 6.88	84.91 ± 5.26	95.09 ± 7.00	87.41 ± 5.28	95.95 ± 6.11	93.95 ± 5.74	56.240	0.001
TG a, mg/dL	252.18 ± 52.81	224.09 ± 47.06	248.36 ± 53.20	222.73 ± 50.39	254.18 ± 56.04	239.09 ± 52.72	9.546	0.001
CHOL a, mg/dL	195.68 ± 34.35	178.64 ± 31.95	193.00 ± 35.69	175.86 ± 31.95	191.68 ± 33/81	181.32 ± 32.44	3.818	0.027
AST a, IU/L	55.0 ± 9.49	37.59 ± 8.67	54.86 ± 11.29	42.5 ± 10.02	56 ± 11.07	37.77 ± 8.78	6.264	0.003
ALT a, IU/L	77.45 ± 18.53	52.27 ± 14.53	78.36 ± 19.88	60.95 ± 14.21	78.73 ± 19.71	53.05 ± 13.99	5.749	0.005
Insulin Levels, mmol/L	14.20 ± 3.78	11.76 ± 3.26	14.17 ± 3.99	12.06 ± 3.57	14.20 ± 3.82	13.50 ± 3.70	28.12	0.001
HOMA-IR a	2.89 ± 0.93	2.12 ± 0.67	2.87 ± 0.98	2.24 ± 0.77	2.9 ± 0.93	2.7 ± 0.85	27.56	0.001

^a^Abbreviations: ALT, alanine aminotransferase; AST, aspartate aminotransferase; BMI, body mass index; CHOL, cholesterol; FBS, fasting blood sugar; HOMA-IR, homeostasis model assessment index for insulin resistance; TG, triglyceride

**Table 3:** Clinical and Non-Clinical Changes of Some Parameters in Three Groups of Intervention

	**FBS** a, **mmol/L**		**TG** a, **mg/dL**		**CHOL** a, **mg/dL**		**ALT** a, **IU/L**		**AST** a, **IU/L**		**HOMA-IR** **a**	
	**Pre No. (%)**	**Post No. (%)**	**Pre No. (%)**	**Post No. (%)**	**Pre No. (%)**	**Post No. (%)**	**Pre No. (%)**	**Post No. (%)**	**Pre No. (%)**	**Post No. (%)**	**Pre No. (%)**	**Post No. (%)**
**Pioglitazone**												
Clinical	7 (31.82)	0 (0)	22 (100)	22 (100)	11 (50)	7 (31/82)	22 (100)	16 (72.73)	22 (100)	7 (31.82)	12 (54.55)	5 (22.73)
Non-clinical	15 (68.18)	22 (100)	0 (0)	0 (0)	11 (50)	15 (68.18)	0 (0)	6 (27.27)	0 (0)	15 (68.18)	10 (45.45)	17 (77.27)
**Metformin**												
Clinical	5 (22.73)	0 (0)	22 (100)	22 (100)	9 (40.91)	6 (27.27)	22 (100)	20 (90.91)	20 (90.91)	10 (45.45)		
	13 (59.1)	8 (36/36)										
Non-clinical	17 (77.27)	22 (100)	0 (0)	0 (0)	13 (59.09)	16 (72.73)	0 (0)	2 (9.09)	2 (9.09)	12 (54.55)	9 (4.9)	14 (63.64)
**Silymarin**												
Clinical	6 (27.27)	3 (13.64)	22 (100)	22 (100)	10 (45.45)	7 (31.8)	22 (100)	18 (81.8)	22 (100)	8 (36.4)	13 (59.1)	13 (59.1)
Non-clinical	16 (72.73)	19 (86.36)	0 (0)	0 (0)	12 (54.55)	5 (68.2)	0 (0)	4 (18.2)	0 (0)	14 (63.6)	9 (40.9)	9 (40.9)
**Total**												
Clinical	18 (72. 7)	3 (4.5)	66 (100)	66 (100)	30 (45.46)	20 (30.3)	66 (100)	54 (81.8)	64 (97)	25 (37.88)	8 (42.4)	20 (30.3)
Non-clinical	48 (27.3)	63 (95.5)	0 (0)	0 (0)	30 (54.55)	46 (69.7)	0 (0)	12 (18.2)	2 (3)	41 (62.12)	28 (54.6)	40 (60.6)

^a^Abbreviations: ALT, alanine aminotransferase; AST, aspartate aminotransferase; CHOL, cholesterol; FBS, fasting blood sugar; HOMA-IR, homeostasis model assessment index for insulin resistance; TG, triglyceride

**Table 4:** Strength of Treatment Effects in Three Groups of Intervention

	**µ1**	**µ2**	**SD**	**Effect Size**
**Pioglitazone**				
Weight, kg	76.95	77.82	9.25	- 0.09
BMI a, kg/m^2^	27.36	27.67	1.68	- 0.18
FBS a, mmol/L	95.45	84.91	6.07	1.74
TG a, mg/dL	252.18	224.09	49.94	0.56
CHOL a, mg/dL	195.68	178.64	33.15	0.51
AST a, IU/L	55.09	37.59	9.08	1.93
ALT a, IU/L	77.45	52.27	16.53	1.52
Insulin Levels, mmol/L	14.20	11.76	3.52	0.69
HOMA-IR a	2.89	2.12	0.80	0.95
**Metformin**				
Weight, kg	76.91	75.46	9.71	0.15
BMI, kg/m^2^	27.53	27.00	1.91	0.28
FBS, mmol/L	95.09	87.41	6.14	1.25
TG, mg/dL	248.36	222.73	51.79	0.49
CHOL, mg/dL	193.00	175.86	33.40	0.51
AST, IU/L	54.86	42.50	10.65	1.16
ALT, IU/L	78.36	60.95	17.04	1.02
Insulin Levels, mmol/L	14.17	12.06	3.78	0.56
HOMA-IR	2.87	2.24	0.87	0.72
**Silymarin**				
Weight, kg	77.00	77.09	9.51	- 0.01
BMI, kg/m^2^	27.44	27.48	1.67	- 0.02
FBS, mmol/L	95.95	93.95	5.93	0.34
TG, mg/dL	254.18	239.09	54.38	0.28
CHOL, mg/dL	191.68	181.32	33.12	0.31
AST, IU/L	56.00	37.77	9.92	1.84
ALT, IU/L	78.73	53.05	16.85	1.52
Insulin Levels, mmol/L	14.19	13.50	3.76	0.18
HOMA-IR	2.90	2.70	0.89	0.22

^a^Abbreviations: ALT, alanineaminotransferase; AST, aspartate aminotransferase;BMI, body mass index; CHOL, cholesterol; FBS, fasting blood sugar;HOMA-IR, homeostasis model assessment index for insulin resistance; TG,triglyceride.

## 5. Discussion

The aim of this study was to assess the effectiveness of silymarin, pioglitazone, and metformin on some biochemical markers in NAFLD. In most studies conducted regarding the NAFLD, the influence of drugs effective in treating this disease has been examined in isolation or together with placebo. In this study the effects of drugs reducing insulin resistance (metformin and pioglitazone) and an antioxidant drug (silymarin) on improvement of biochemical indices, weight, and body mass index were compared. These three drugs showed beneficial effects on NAFLD treatment and no specific side effects were reported in relation to their uptake. Given the fact that it seems NASH is a part of metabolic syndrome, the effects of these drugs on lipid profile, blood sugar, serum insulin level, and HOMA Index were also examined in addition to their effects on decrease in hepatic aminotransferases. In this study, significant differences were observed between average biochemical indices before and after intervention (drug consumption) in patients with NASH in all three treatment groups. Metformin and pioglitazone exhibited large effects on FBS, and moderate effects on TG, cholesterol, and Insulin. All drugs had large effects on ALT and AST. In a Meta-analysis study to assess Metformin, eleven RCTs (671 participants, 27% diabetic; six RCTs in NASH with post-treatment histology, three with a low bias risk), the results showed that anorexigenic and weight-loss, decreases gastrointestinal glucose absorption and increases insulin sensitivity, and AMP kinase-mediated oxidative glucose and lipid metabolism have been accrued in these patients. Liver histology compared to placebo was not improved but body weight, waist circumference, HOMA, FBS, HbA1c, increased HDL-C, and adiponectin significantly reduced (11). In one study, the effects of pioglitazone and placebo were examined on patients with NASH for a 12-month period and weight increase was observed in the group being treated with pioglitazone (12). In a Meta-analysis study about pioglitazone, results showed that pioglitazone did not further improve treatment of any diabetic patient with NASH, fatty liver, and liver histology. However HOMA and transaminases improved over two and three years, respectively (11). In present study a significant decrease was observed in mean fasting blood glucose and serum insulin level in all groups, and also differences in average changes were significant between the groups. The most reduction in blood glucose level was observed in pioglitazone group. The least decrease in blood sugar was observed in silymarin group and the difference in average changes was significant between this and two other groups. In one study, there was no significant decrease in fasting blood glucose level before and after treatment with silymarin compared to placebo (13). However, in another study, silymarin was associated with decrease in resistance to insulin and serum insulin level in patients with cirrhosis and diabetes (14). Given the role played by pioglitazone and metformin in treatment of diabetes, results obtained from the study were expectable but the noticeable point was the effect of silymarin on decreasing blood sugar and insulin level, and HOMA index.

In this study a significant reduction in serum cholesterol and triglyceride was observed in all three treatment groups. The most reduction in triglyceride was observed in pioglitazone group and the least average change was observed in silymarin group. The most reduction in cholesterol was observed in metformin group and difference in average changes in cholesterol also was not significant between pioglitazone and silymarin groups. In one study in Miami University of U.S.A comparing pioglitazone and placebo effects in patients with NASH, no changes were observed in lipid profile after the intervention (1). In the present study, decrease in AST and ALT levels was observed in all three groups but in silymarin group it was more than that of other groups. In previous studies, decrease in hepatic transaminases was reported after taking silymarin (13, 15, 16). In some studies on metformin and pioglitazone, decrease in hepatic transaminases level and histological improvement were reported in patients with NASH. In present study patients tolerated the drugs very well, and except for weight gain, no specific disorders were observed in relation to pioglitazone drug, although due to cardiac side effect it is not an issue for long term treatment of NAFLD. The probability of weight gain increases with long-term consumption of pioglitazone which can lead to weakening of its beneficial effects on treatment of NAFLD and it seems that more studies in this field are necessary to conduct before recommending this drug. In summary, results show that all drugs decrease ALT and AST but pioglitazone and metformin can reduce lipid profile as well as improve insulin and glucose parameters. Moreover, Silymarin is well tolerated and seems to be with no side effects when used for two months. Given the increasing incidence of non-alcoholic fatty liver disease and its subsequent outcomes in the form of cirrhosis and hepatocellular carcinoma and given the relationship between this disease and increase in resistance to insulin and oxidative stress, it seems necessary to conduct longer period studies on silymarin, metformin, and pioglitazone, and examine the effects of other antioxidants and drugs reducing resistance to insulin.

## References

[1] Al-Gharabally A, O’Brien CB, Acosta RC (2007). A pilot study of pioglitazon for treatment of non-alcholic fatty liver disease.. Hepat Mon.

[2] Serfaty L, Lemoine M (2008). Definition and natural history of metabolic steatosis: clinical aspects of NAFLD, NASH and cirrhosis.. Diabetes Metab.

[3] Farrell GC, van Rooyen D, Gan L, Chitturi S (2012). NASH is an Inflammatory Disorder: Pathogenic, Prognostic and Therapeutic Implications.. Gut Liver.

[4] Glueck CJ, Moreira A, Goldenberg N, Sieve L, Wang P (2003). Pioglitazone and metformin in obese women with polycystic ovary syndrome not optimally responsive to metformin.. Hum Reprod.

[5] Haas DA, Carr BR, Attia GR (2003). Effects of metformin on body mass index, menstrual cyclicity, and ovulation induction in women with polycystic ovary syndrome.. Fertil Steril.

[6] Harborne L, Fleming R, Lyall H, Norman J, Sattar N (2003). Descriptive review of the evidence for the use of metformin in polycystic ovary syndrome.. Lancet.

[7] Katsiki N, Hatzitolios AI (2010). Insulin-sensitizing agents in the treatment of polycystic ovary syndrome: an update.. Curr Opin Obstet Gynecol.

[8] Lord JM, Flight IH, Norman RJ (2003). Insulin-sensitising drugs (metformin, troglitazone, rosiglitazone, pioglitazone, D-chiro-inositol) for polycystic ovary syndrome.. Cochrane Database Syst Rev.

[9] Cho LW, Kilpatrick ES, Keevil BG, Coady AM, Atkin SL (2009). Effect of metformin, orlistat and pioglitazone treatment on mean insulin resistance and its biological variability in polycystic ovary syndrome.. Clin Endocrinol (Oxf)..

[10] Ortega-Gonzalez C, Luna S, Hernandez L, Crespo G, Aguayo P, Arteaga-Troncoso G (2005). Responses of serum androgen and insulin resistance to metformin and pioglitazone in obese, insulin-resistant women with polycystic ovary syndrome.. J Clin Endocrinol Metab.

[11] Musso G, Cassader M, Rosina F, Gambino R (2012). Impact of current treatments on liver disease, glucose metabolism and cardiovascular risk in non-alcoholic fatty liver disease (NAFLD): a systematic review and meta-analysis of randomised trials.. Diabetologia.

[12] Aithal GP, Thomas JA, Kaye PV, Lawson A, Ryder SD, Spendlove I (2008). Randomized, placebo-controlled trial of pioglitazone in nondiabetic subjects with nonalcoholic steatohepatitis.. Gastroenterology.

[13] Hashemi SJ, Hajiani E, Haidari Sardabi E (2009). A placebo-controlled trial of silymarin in patients with nonalcoholic fatty liver disease.. Hepat Mon.

[14] Velussi M, Cernigoi AM, De Monte A, Dapas F, Caffau C, Zilli M (1997). Long-term (12 months) treatment with an anti-oxidant drug (silymarin) is effective on hyperinsulinemia, exogenous insulin need and malondialdehyde levels in cirrhotic diabetic patients.. J Hepatol.

[15] Federico A, Trappoliere M, Tuccillo C, de Sio I, Di Leva A, Del Vecchio, Blanco C (2006). A new silybin-vitamin E-phospholipid complex improves insulin resistance and liver damage in patients with non-alcoholic fatty liver disease: preliminary observations.. Gut.

[16] Hajaghamohammadi AA, Ziaee A, Rafiei R (2008). The efficacy of silymarin in decreasing transaminase activities in non-alcoholic fatty liver disease: A randomized controlled clinical tria.. Hepat Mon.

